# Perceptions regarding the Indian Mental Healthcare Act 2017 among psychiatrists: Review and critical appraisal in the light of CRPD guidelines

**DOI:** 10.1017/gmh.2024.31

**Published:** 2024-03-19

**Authors:** N.A. Uvais, Kaustubh Joag

**Affiliations:** 1 Department of Psychiatry, Iqraa International Hospital and Research Centre, Calicut, India; 2 Centre for Mental Health Law & Policy, Indian Law Society (ILS), Pune, India

**Keywords:** Indian Mental Healthcare Act 2017, perception, psychiatrists, CRPD

## Abstract

**Background:**

Informed by the UN Convention on the Rights of Persons with Disabilities, the Indian government replaced the 1987 Mental Health Act with the transformative “Indian Mental Healthcare Act, 2017” (IMHCA 2017), which gained presidential approval on April 7, 2017. While the new act aligns with CRPD guidelines, emphasizing the promotion, protection and realization of complete and equitable human rights, legal capacity, equality and dignity for persons with mental illness, it has faced diverse criticism from various stakeholders, particularly psychiatrists. This study systematically explores the critiques and apprehensions expressed by psychiatrists regarding the IMHCA 2017 using available published resources and assesses these criticisms within the context of CRPD guidelines.

**Methodology:**

We conducted a scoping review of the literature, using two search engines like PubMed and Scopus. The review covered academic publications, reports and documents from both national and international sources, authored by psychiatrists and psychiatric organizations, related to the IMHCA 2017. The primary search term “IMHCA 2017” was used without temporal restrictions. Publications authored by mental health professionals from India and around the world were included in the final analysis. Through qualitative analysis, key themes reflecting psychiatrists’ viewpoints were identified. These themes, marked by substantial criticism, were then assessed in accordance with the guiding principles of the CRPD, including its optional protocol and general comments.

**Results:**

The study analyzed 33 manuscripts discussing criticisms and concerns about IMHCA 2017. Manuscript types included opinion papers (60.6%), original research articles (21.21%), review articles (9.09%), editorials (6.06%) and comments (3.03%). All but one article were authored by psychiatrists, with five by non-Indian authors and the rest by Indian psychiatrists. Most articles were published in the Indian Journal of Psychiatry (75.76%), with some in other journals. About 54.55% critically scrutinized act provisions, while 45.45% highlighted positive aspects. The analysis identified seven prominent criticism themes: clinical apprehensions, lack of clarity and comprehensiveness, feasibility challenges, neglect of caregivers, mistrust toward psychiatrists, crises in general hospital psychiatry units and ideological reservations.

**Conclusions:**

Each theme was critically assessed in the context of CRPD guidelines, and corresponding recommendations were formulated.

## Impact statement

This article carries significant implications for mental health policy and practice. By elucidating psychiatrists’ multifaceted criticisms through the lens of Convention on the Rights of Persons with Disabilities (CRPD) guidelines, the study facilitates a nuanced understanding of challenges in implementing the Indian Mental Healthcare Act (IMHCA) 2017. The identified themes provide policymakers with targeted insights, offering a roadmap for refining mental health legislation and addressing concerns raised by frontline mental health practitioners. The impact extends to the broader healthcare landscape, emphasizing the need for a collaborative approach that engages psychiatrists in the ongoing evolution of mental health services. Furthermore, the study underscores the importance of aligning mental health policies with international human rights standards, particularly the CRPD, ensuring the rights and dignity of individuals with mental health conditions. Beyond academia, the article informs mental health professionals, policymakers and advocacy groups, fostering awareness about the complexities of implementing a rights-based approach. It calls for ongoing dialog, education and training initiatives to bridge the gap between policy intentions and on-the-ground realities, promoting a patient-centric and ethically sound mental healthcare system in India.

## Background

Mental health holds significant importance in India’s public health landscape, with approximately 0.8% of the population facing notable mental health challenges (Gururaj et al., 2016). Studies reveal that persons with mental illness (PwMI) frequently endure human rights violations, resulting in societal marginalization due to associated stigma and discrimination (Mfoafo-M’Carthy and Huls, 2014). This underscores the urgent need to uphold, advocate for and safeguard the rights of PwMIs (Mfoafo-M’Carthy and Huls, 2014). The UN Convention on the Rights of Persons with Disabilities (UNCRPD), established on December 13, 2006, aims to ensure the complete and equal enjoyment of human rights and freedoms for all individuals with disabilities, fostering respect for their inherent dignity (Mfoafo-M’Carthy and Huls, 2014). India signed the UNCRPD on March 30, 2007, and formalized its commitment by ratifying the CRPD on October 1, 2007. This ratification underscored India’s dedication to upholding the rights and dignity of persons with disabilities as articulated in the CRPD. The impact of this ratification extended beyond influencing the development of the Mental Healthcare Act 2017; it catalyzed the advancement of inclusive services and fostered a rights-based attitude toward mental health. This transformation was evident in increased awareness, sensitization efforts and legal empowerment initiatives, marking a significant shift in how mental health and disability are perceived in India (Chaturvedi et al., 2015).

A pivotal element in enhancing mental healthcare in India was the reform of mental health legislation. Before the enactment of the Indian Mental Healthcare Act (IMHCA) in 2017, mental healthcare in India encountered several challenges. The mental health landscape suffered from the absence of a comprehensive legal framework, leading to a lack of clear guidelines for the rights and treatment of individuals with mental health conditions. The existing laws, primarily embodied in the Mental Health Act (MHA) of 1987, were outdated and did not align with contemporary international standards. These laws focused more on custodial care rather than promoting the rights and autonomy of individuals with mental illnesses.

Furthermore, pervasive stigma and discrimination against people living with mental illnesses created barriers to help seeking, treatment and rehabilitation. Treatment predominantly occurred in larger institutions, neglecting the need for community-based services. Involuntary admissions and seclusions of individuals with mental illnesses were common, as the existing law allowed such practices, resulting in poor safeguards for patient rights. Additionally, there was limited accessibility and awareness of mental health services, particularly in rural India.

The acceptance of the MHA of 1987 among psychiatrists in India exhibited a range of perspectives. While some practitioners acknowledged the necessity of having a legal framework to regulate mental healthcare, others voiced reservations and criticisms regarding the act’s effectiveness and appropriateness. Key concerns included issues related to custodial care, a lack of clarity concerning the rights and treatment of individuals with mental health conditions, involuntary admissions and seclusions and the perpetuation of stigma.

Aligned with the principles of the UNCRPD, the Indian government enacted the progressive IMHCA 2017, which received presidential approval on April 7, 2017 (Chaturvedi et al., 2015). IMHCA 2017 aimed to rectify these issues by introducing a rights-based approach, advocating for community-based care, safeguarding patient rights and aligning with international standards outlined in the CRPD. The enactment of IMHCA marked a crucial step toward transforming the mental health landscape in India, emphasizing dignity, autonomy and inclusivity for individuals with mental health conditions (Mishra and Galhotra, 2018). Apart from CRPD, the IMHCA 2017 was also influenced by national and international human right standards, public advocacy and stakeholder involvement, recommendations from mental health professionals, research data and reports, global trends and best practices and judicial decisions.

While the new act aligns with CRPD guidelines, emphasizing the promotion, protection and realization of complete and equitable human rights, legal capacity, equality and dignity for PwMI, it has faced diverse criticism from various stakeholders, particularly psychiatrists (Duffy et al., 2019a, 2019b; Korulla, 2019). Despite the progressive nature of the IMHCA, its implementation has encountered challenges. Community-based services have not been fully realized, and mental health programs may still be concentrated in larger institutions. Involuntary admissions and seclusions persist to some extent, and safeguards for patient rights are not consistently enforced. Engaging psychiatrists is crucial to bridge the gap between policy and implementation. Psychiatrists play a pivotal role in shaping mental health services, and their active involvement is necessary for the success of community-based care. The attitude and acceptance are crucial elements for the successful implementation of the IMHCA 2017. A positive and receptive attitude among healthcare professionals, including psychiatrists, is essential to ensure the effective execution of the provisions outlined in the act. Acceptance of the act’s principles, such as a rights-based approach and community-based care, is pivotal for mental health professionals to align their practices with the legislative framework. The active involvement and support of stakeholders, including psychiatrists, play a key role in translating the legal guidelines into practical, patient-centric mental health services. Their involvement in the policy-making process is crucial, necessitating careful consideration of their concerns. Indian mental health professionals, particularly psychiatrists, have authored publications critiquing the act on multiple fronts, encompassing feasibility, clinical aspects, cultural nuances and more (Duffy et al., 2019a, 2019b; Korulla, 2019). A systematic exploration of these critiques is vital for informing policy decisions (Duffy et al., 2019a, 2019b; Korulla, 2019). Furthermore, targeted educational initiatives can enhance psychiatrists’ understanding of the act’s underlying principles, potentially fostering greater appreciation for its significance. By engaging psychiatrists and addressing their perspectives, the act’s acceptance may improve, facilitating smoother implementation of mental health programs grounded in the act and benefiting from their wholehearted collaboration.

The purpose of this study is to comprehensively investigate and analyze the critiques expressed by psychiatrists regarding the IMHCA 2017. By delving into the available published resources, the study seeks to identify the specific areas of concern, challenges and reservations that psychiatrists may have regarding the implementation and implications of the IMHCA. The need for this study arises from the recognition that psychiatrists, as frontline mental health practitioners, play a pivotal role in the effective execution of mental health policies. Their perspectives and reservations are critical in shaping the landscape of mental healthcare in India. By assessing the criticisms within the context of CRPD guidelines, the study aims to contribute valuable insights into potential barriers and areas for improvement in the IMHCA. Addressing these concerns is essential for fostering a collaborative approach between policymakers, mental health professionals and other stakeholders, ensuring that the legislation achieves its intended goals while upholding the rights and well-being of individuals with mental health conditions.

## Methodology

A scoping review methodology was employed to synthesize relevant information from published studies addressing the perceptions of psychiatrists regarding the IMHCA 2017. The review adhered to the Preferred Reporting Items for Systematic Reviews and Meta-Analyses (PRISMA) checklist.

## Literature search

The search encompassed national and international journals, as well as reports and documents from gray literature. The focus was on works authored by psychiatrists and psychiatric organizations. Inclusion and exclusion criteria were defined to guide the selection process. Various search strategies were applied across two major databases to ensure thorough exploration of the available literature. The exploration of gray literature in this review encompassed unconventional sources like reports, documents and publications beyond traditional academic journals. Specific strategies involved accessing conference proceedings and books, ensuring a comprehensive approach to gather diverse perspectives on the topic.

## Eligibility criteria

The review included studies focusing specifically on the perceptions and experiences of psychiatrists regarding IMHCA 2017. Inclusion criteria comprised publications describing these perceptions, published in the English language.

## Electronic database searching

PubMed and Scopus were utilized to obtain relevant literature, utilizing the primary keyword “IMHCA 2017” with no temporal restrictions.

## Data extraction and quality assessment

**Figure 1.**
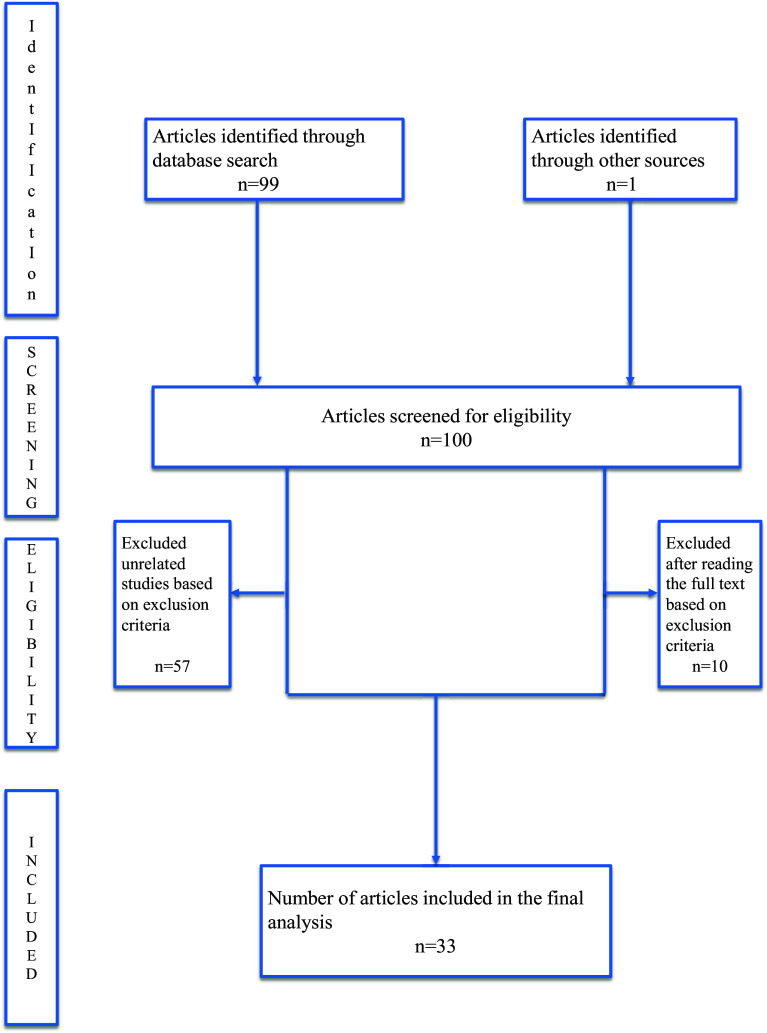
PRISMA flow diagram showing the selection of studies for the review.

A total of 99 references were identified through the literature search. Articles were evaluated for relevance, appropriateness and clarity. The PRISMA flow diagram guided the selection process, resulting in the inclusion of 33 studies for this review (Figure 1).

## Data analysis

1. *Preliminary organization of studies*: Key data, including Author, Journal, Year, Country and manuscript type, were tabulated from the selected articles.
2. *Categorizing studies based on review objectives*: The studies were examined to gather data aligned with the predefined review objectives.
3. *Summarizing major findings*: Descriptive statistics facilitated the analysis and summarization of data.
4. *Synthesis of data*: Data synthesis involved identifying common themes in published literature related to psychiatrists’ perceptions of IMHCA 2017.
5. *Critical appraisal in light of CRPD guidelines*: The identified themes, marked by significant critique, underwent a critical evaluation. This assessment was conducted in alignment with the guiding principles of the CRPD, including its optional protocol and general comments.

## Results

The final analysis of this study encompassed 33 manuscripts that discussed criticisms and/or concerns related to IMHCA 2017. The predominant manuscript types were opinion papers (60.6%), followed by original research articles (21.21%), review articles (9.09%), editorials (6.06%) and comments (3.03%). With a single exception, all articles were authored by psychiatrists; among these, five were written by non-Indian authors, while the remainder were penned by Indian psychiatrists. Overall, 54.55% of the manuscripts critically scrutinized various provisions of the act, while 45.45% highlighted positive aspects of its provisions. The details of the journals in which the articles were published are summarized in Table 1.

**Table 1.** Details of journals

Name	% of articles
Indian Journal of Psychiatry	75.76%
Indian Journal of Psychological Medicine	9.09%
Indian Journal of Medical Ethics	3.03%
International Journal of Law and Psychiatry	3.03%
International Journal of Mental Health Systems	3.03%
Journal of Public Mental Health	3.03%
Educere – BCM Journal of Social Work	3.03%

Through an analysis of the selected literature, seven prominent themes of criticisms targeting IMHCA 2017 by psychiatrists emerged. The most prevalent theme pertained to clinical apprehensions associated with the act’s execution. Additional recurrent themes included concerns about lack of clarity and comprehensiveness and feasibility challenges tied to implementing the act. Other identified themes encompassed neglect of caregivers, the promotion of mistrust toward psychiatrists, the potential creation of crises in general hospital psychiatry units and ideological reservations. The summarized findings are presented in Table 2.

**Table 2.** Summarizing the themes of criticisms in manuscripts

	Authors	Written by	Type of the manuscript	Position regarding MHA 2017	Criticism
1	Ameen et al., 2019	Psychiatrists	Opinion	Positive	Feasibility concerns
2	Harbishettar et al., 2019a	Psychiatrists	Opinion	Positive	Feasibility concerns
3	Prashanth et al., 2019	Psychiatrists	Review	Positive	Feasibility concerns
4	Harbishettar et al., 2019a	Psychiatrists	Review	Critical	Clinical concerns
5	Kapoor and Pathare, 2019	Research associate and psychiatrist	Comment	Positive	Nil
6	Jagadish et al., 2019	Psychiatrist	Editorial	Critical	Lack of clarity and comprehensiveness Lack of concern for the caregivers Promotes mistrust toward clinicians
7	Math et al., 2019a	Psychiatrist	Original Research	Positive	Feasibility concerns
8	Pavitra et al., 2019	Psychiatrist	Opinion	Critical	Lack of concern for the caregivers
9	Mohan and Math, 2019	Psychiatrists	Opinion	Positive	Lack of clarity and comprehensiveness
10	Math et al., 2019	Psychiatrists	Opinion	Critical	Clinical concerns Promotes mistrust toward clinicians Lack of concern for the caregivers Lack of clarity and comprehensiveness Ideological concerns
11	Harbishettar and Murthy, 2019a	Psychiatrists	Opinion	Positive	Feasibility concerns
12	Sharma and Kommu, 2019	Psychiatrists	Opinion	Positive	Lack of clarity and comprehensiveness
13	Namboodiri, 2019	psychiatrist	Opinion	Critical	Clinical concerns.
14	Vadlamani and Gowda, 2019	Psychiatrists	Opinion	Positive	Clinical concerns
15	Duffy and Kelly, 2019a	Psychiatrists	Original article	Positive	Feasibility concerns Clinical concerns Promotes mistrust toward clinicians Lack of concern for the caregivers Lack of clarity and comprehensiveness Ideological concerns
16	Mannekote et al., 2019	Psychiatrists	Review	Positive	Clinical concerns
17	Rao et al., 2019	Psychiatrists	Opinion	Critical	Lack of clarity and comprehensiveness
18	Swaminath et al., 2019	Psychiatrists	Opinion	Positive	Clinical concerns
19	Duffy et al., 2019a	Psychiatrists	Original research	Critical	Feasibility concerns Clinical concerns
20	Nallur, 2019	Psychiatrist	Opinion	Positive	Lack of clarity and comprehensiveness
21	Sivakumar et al., 2019	Psychiatrists	Opinion	Critical	Clinical concerns Lack of concern for the caregivers Lack of clarity and comprehensiveness
22	Duffy and Kelly, 2017	Psychiatrists	Original Research	Positive	Lack of concern for the caregivers Lack of clarity and comprehensiveness
23	Duffy et al., 2018	Psychiatrists	Original research	Positive	Feasibility concerns Clinical concerns Bad for general hospital psychiatric units
24	Kumar, 2018	Psychiatrist	Opinion	Critical	Clinical concerns Lack of concern for the caregivers Bad for general hospital psychiatric units
25	Singh, 2019	Psychiatrist	Editorial	Critical	Feasibility concerns Lack of clarity and comprehensiveness Bad for general hospital psychiatric units
26	Philip et al., 2019	Psychiatrists	Opinion	Critical	Clinical concerns
27	Bijal et al., 2019	Psychiatrists	Opinion	Positive	Feasibility concerns
28	Kumar et al., 2019	Psychiatrists	Opinion	Positive	Lack of clarity and comprehensiveness
29	Raveesh et al., 2019	Psychiatrists	Opinion	Critical	Lack of clarity and comprehensiveness
30	Gowda et al., 2019	Psychiatrists	Opinion	Critical	Feasibility concerns Clinical concerns Promotes mistrust toward clinicians Lack of concern for the caregivers
31	Ali et al., 2019	Psychiatrists	Opinion	Critical	Feasibility concerns Clinical concerns Lack of clarity and comprehensiveness
32	Duffy et al., 2019a	Psychiatrists	Original Research	Critical	Lack of clarity and comprehensiveness Bad for general hospital psychiatric units
33	Korulla, 2019	Social worker	Original research	Critical	Feasibility concerns Clinical concerns Promotes mistrust toward clinicians

## Concerns related to clinical practice

A significant concern voiced by psychiatrists regarding IMHCA 2017 pertains to its potential negative impact on routine psychiatric practice. These clinical concerns can be categorized into three main domains: admissions, treatments and decision-making processes.

1. *Admission-related concerns*: IMHCA 2017 delineates stringent criteria for involuntary admission to mental healthcare facilities, hinging on capacity evaluation and the degree of potential harm to oneself or others. Section 89 of IMHCA 2017 specifies that supported admission upon a nominated representative’s request must follow an independent assessment by a psychiatrist and a mental health professional or medical practitioner to ascertain immediate risk. Numerous psychiatrists contend that adhering to such stringent criteria could conflict with their patients’ best interests (Bijal et al., 2019; Harbishettar et al., 2019b; Math et al., 2019b; Namboodiri, 2019; Vadlamani and Gowda, 2019). They point out that patients with severe mental illnesses or substance use disorders, who may necessitate inpatient care based on psychiatric evaluations, might be ineligible for involuntary admission due to retained capacity or nonthreatening psychiatric symptoms. This, in turn, could contribute to a rise in homeless individuals with mental health issues, crime and violence. However, it is important to note that some of these concerns are subjective and lack substantial evidence to support them.
2. *Treatment-related concerns:* The IMHCA 2017 incorporates provisions that necessitate meticulous documentation of assessments and treatment outcomes by mental health professionals to safeguard the rights of individuals with mental illness. However, numerous psychiatrists express concern that the increased time spent on administrative tasks and detailed documentation might curtail valuable patient engagement for clinical discussions (Duffy and Kelly, 2019a; Duffy et al., 2019b; Harbishettar et al., 2019b).

Sub-section 5 of Section 86 within IMHCA 2017 strictly prohibits administering psychiatric treatment to an autonomous patient without their informed consent. Many psychiatrists are apprehensive that upholding patients’ rights to refuse treatment could potentially amplify dropout rates, subsequently leading to relapses and rehospitalizations (Andrade et al., 2003; Bijal et al., 2019; Harbishettar et al., 2019b; Namboodiri, 2019).

Section 5 of IMHCA 2017 outlines the right for every non-minor individual to create a written advance directive, specifying preferences for mental healthcare and treatment. As stipulated in Section 10, healthcare providers are obliged to offer treatment in alignment with valid advance directives. Section 11 mandates that any modifications or cancelations to advance directives must involve an application to the pertinent board through a healthcare provider. Some psychiatrists contend that delivering mental healthcare to suicide attempt survivors may pose significant challenges, particularly when an advance directive under IMHCA 2017 is in place. Revoking such directives necessitates engagement with the mental health review board, potentially introducing barriers and delays due to resource constraints (Demarco, 2005; Korulla, 2019; Kumar et al., 2019a, 2019b; Vadlamani and Gowda, 2019).

Additionally, psychiatrists express notable concerns regarding the regulation of electroconvulsive therapy (ECT). Section 95 of IMHCA 2017 prohibits unmodified ECT, emergency ECT and ECT for minors, a decision met with skepticism by many psychiatrists who believe this could deprive patients of a potentially life-saving psychiatric intervention (Ali et al., 2019; Duffy et al., 2019a; Math et al., 2019b).

3. *Decision-making-related concerns*: The IMHCA 2017 encompasses a range of provisions aimed at safeguarding the rights of PwMI, including Sections 88 (voluntary discharge), 5 and 10 (Advance Directive), sub-section 5 of Section 86 (prohibiting involuntary treatment of independent patients), Section 14 (Nominated Representative) and Section 73 (Mental Health Review Boards), among others. However, certain psychiatrists argue that these provisions fail to account for the positive impact of the therapeutic alliance between patients and clinicians, potentially fostering a climate of defensive medicine where practitioners undertake treatments or procedures to mitigate the risk of malpractice litigation (Duffy and Kelly, 2019a; Harbishettar et al., 2019b; Math et al., 2019b). Furthermore, concerns arise that the involvement of nominated representatives and mental health review boards may shift decision-making power away from mental health professionals to individuals lacking expertise in the field (Bijal et al., 2019; Duffy and Kelly, 2019a).

## Lack of clarity and comprehensiveness

An essential critique raised by psychiatrists against the IMHCA 2017 revolves around its lack of clarity and comprehensiveness. According to these experts, this ambiguity commences with the act’s definition of mental illness (Duffy and Kelly, 2019a; Duffy et al., 2019a; Math et al., 2019b). The act defines mental illness as “a substantial disorder of thinking, mood, perception, orientation or memory that grossly impairs judgment, behavior, capacity to recognize reality or ability to meet the ordinary demands of life, mental conditions associated with the abuse of alcohol and drugs, but does not include mental retardation which is a condition of arrested or incomplete development of mind of a person, specially characterized by subnormality of intelligence.” This definition, however, omits certain psychiatric conditions that may not lead to significant cognitive alteration but still cause considerable distress and dysfunction, such as common mental disorders or harmful substance use (Rugkåsa and Canvin, 2017; Mohan and Math, 2019; Raveesh et al., 2019). Furthermore, neurodevelopmental disorders, which often manifest with psychiatric symptoms, are not encompassed by this definition, possibly leading to confusion and treatment dilemmas (Bijal et al., 2019; Sharma and Kommu, 2019).

Section 4 of the IMHCA 2017 asserts that every individual, including PwMI, is presumed to possess the capacity to make decisions about their mental healthcare or treatment provided they can understand relevant information, appreciate foreseeable consequences and communicate their decisions. However, the vagueness of this mental health capacity definition, without explicit mention of legal capacity, raises concerns (Jagadish et al., 2019; Kumar et al., 2019a, 2019b; Raveesh et al., 2019). Without well-defined criteria to assess mental capacity and the absence of legal capacity, as enshrined in Article 12 of the CRPD, coercive treatments involving nominated representatives could become prevalent. Additionally, the act does not sufficiently address capacity assessment for treatment decisions. Literal interpretation of the act’s “harm to self or others” concept could lead to involuntary admissions for many individuals with substance use disorders, and the absence of a “Code of Practice” may foster differing interpretations, creating confusion among stakeholders (Bijal et al., 2019). The act also lacks clarity in the process of selecting nominated representatives during family disputes (Kumar et al., 2019a).

Furthermore, the act is perceived as lacking comprehensive coverage by several psychiatrists, as it remains silent on community-based care for patients with mental illness, issues related to social rights, stigma and discrimination (Prashanth et al., 2019; Sharma and Kommu, 2019). Although Section 18 of the IMHCA 2017 outlines patient rights, these provisions do not directly address various challenges faced by PwMI, such as social rights and discrimination, with many rights described in the CRPD absent from the IMHCA 2017.

The act’s provisions also fail to address the potential utilization of technology or provide a framework for leveraging it, despite many psychiatrists considering technological solutions as a viable remedy for India’s significant mental health workforce shortage (Singh, 2019). The omission of guidelines and regulations for digital psychiatry is perceived as a significant gap in the IMHCA 2017.

Finally, some psychiatrists express concern that the act does not adequately address the concept of vulnerability, which holds central importance in the realm of mental healthcare (Philip et al., 2019).

## Feasibility concerns with implementation of the act

Another substantial critique voiced by psychiatrists against the MHA 2017 pertained to its feasibility. Many raised concerns about the practical implementation of various provisions of the act, taking into account factors such as work culture, limited human resources, political support, available funding and existing infrastructure (Kumar, 2018; Ameen et al., 2019; Bijal et al., 2019; Duffy and Kelly, 2019a; Duffy et al., 2019a, 2019b; Harbishettar and Murthy, 2019a; Harbishettar et al., 2019a; Korulla, 2019; Kumar et al., 2019b; Math et al., 2019a; Prashanth et al., 2019). Some psychiatrists specifically highlighted reservations about particular aspects of the act. For instance, sub-section 5 of Section 86 mandates that any mental health intervention on an independent patient must only proceed with informed consent. A faction of psychiatrists apprehended that conducting an informed consent process before each psychiatric intervention might not be viable given the scarcity of psychiatrists and time constraints due to the high patient volume (Harbishettar et al., 2019a; Prashanth et al., 2019). In contrast, another group expressed worry that stipulations requiring capacity assessment (Section 4 of IMHCA 2017) and adherence to advance directives (Sections 5 and 10) could escalate the cost of psychiatric treatment (Kumar, 2018; Bijal et al., 2019; Kumar et al., 2019b). Certain critics also contested the introduction of advance directives in the Indian context, citing a lack of evidence-based support (Duffy and Kelly, 2019a). Additionally, many psychiatrists perceived the mental health review board (Section 73 of IMHCA 2017) as an impediment to the delivery of mental healthcare (Bijal et al., 2019; Kumar et al., 2019b). They argued that procedural requirements could delay urgent psychiatric care, due to resource shortages, which is in the best interest of the patient. Furthermore, concerns were raised about the impracticality of obtaining informed consent from nominated representatives in emergency psychiatric situations (Kumar et al., 2019b).

## Lack of concern for the caregivers

Another significant criticism directed at the IMHCA 2017 pertained to its perceived lack of emphasis on the role of caregivers for individuals living with mental health issues. Concerns were raised by certain psychiatrists that certain provisions within the act, particularly those allowing for nominated representatives beyond immediate family, might inadvertently foster an incorrect perception that family members are the primary violators of the human rights of those with mental health conditions. This, in turn, could potentially disrupt family dynamics (Ali et al., 2019; Bijal et al., 2019; Duffy and Kelly, 2019a; Jagadish et al., 2019; Math et al., 2019b; Pavitra et al., 2019; Raveesh et al., 2019). Some psychiatrists also expressed the view that the provision of advance directives could lead to increased treatment costs, thereby placing an additional financial burden on families (Jagadish et al., 2019). Additionally, reservations were expressed regarding the lack of clarity surrounding the selection of nominated representatives in cases where disputes arise among family members regarding the responsibility of caring for older adults (Kumar et al., 2019a).

Many psychiatrists believed that the IMHCA 2017 did not adequately acknowledge the pivotal role of family members and caregivers as primary stakeholders in supporting individuals with mental health challenges. The act was seen to overlook caregivers’ burdens, the isolation they experience and the frustration they endure (Ali et al., 2019; Bijal et al., 2019; Raveesh et al., 2019). Furthermore, the IMHCA 2017 was deemed insufficient in addressing the rights (such as the right to information, treatment planning or appeal) and the caregiving burdens borne by those supporting individuals with mental health issues (Bijal et al., 2019). By not adequately recognizing and reflecting the essential role of family, the act was thought to undermine the rights of families and caregivers in their vital support of individuals living with mental health conditions.

## Promotes mistrust toward clinicians

Another notable critique put forth by psychiatrists concerning IMHCA 2017 is that certain provisions of the act might inadvertently foster a sense of mistrust toward clinicians among patients and society at large. This criticism stems from the fact that the Indian Psychiatric Society, the leading professional organization representing psychiatrists in India, was not involved in the drafting process of the act, a contrast to the approach taken during the development of the previous MHA, 1987 (Jagadish et al., 2019). Some psychiatrists argued that the act’s emphasis on a rights-based approach, spotlighting individual patient autonomy in decision-making through measures like nominated representatives and advanced directives, could potentially undermine the role of clinicians in providing care and support to individuals with mental illness (Bijal et al., 2019; Math et al., 2019b).

Moreover, certain psychiatrists held the view that the provisions of the act underwent a fundamental transformation of mental healthcare from a medical paradigm to a more legal framework. This shift, they contended, diluted the positive impact of the doctor–patient relationship in delivering mental healthcare and inadvertently encouraged defensive medical practices (Duffy and Kelly, 2019a).

Additionally, some psychiatrists raised the concern that the act’s new provisions, such as nominated representatives, advanced directives and mental health review boards, might create the impression that clinicians are the primary violators of the rights of individuals with mental illness (Math et al., 2019b). Furthermore, these provisions might be seen as shifting mental healthcare decision-making away from experts to non-experts, potentially eroding the role of clinicians in this crucial process (Duffy and Kelly, 2019a; Korulla, 2019).

## Creates crisis for general hospital psychiatric units (GHPU)

Another crucial criticism posed by psychiatrists regarding IMHCA 2017 revolves around its potential inadequacy in promoting the development of GHPUs. Section 65 of IMHCA 2017 stipulates that treating patients in unregistered mental health establishments is prohibited. Many of the GHPUs in India function solely under the hospital license and lack specific registration under the mental healthcare act. Certain psychiatrists express concerns that this mandatory certification requirement for mental health establishments and inpatient care of psychiatric patients could create legal obstacles in providing treatment within GHPUs (Ali et al., 2019; Duffy et al., 2019a, 2019b; Raveesh et al., 2019). There are also fears that classifying patients based on mental health issues in general hospitals and subsequently transferring them to certified mental health establishments for further treatment, as mandated by IMHCA 2017, might compel individuals with mental illness to seek treatment from stand-alone psychiatric hospitals. This, in turn, might exacerbate stigma and discrimination (Duffy et al., 2019b).

Furthermore, some psychiatrists argue that by encompassing all voluntary admissions of adult individuals with mental health issues under its purview, the act may impede the ability of mentally competent adults to be admitted to general hospitals for treatment similar to physical illnesses. Such a provision could potentially be perceived as a violation of the individual autonomy of persons with mental health problems (Ali et al., 2019).

Finally, concerns are raised that the act’s potential promotion of stand-alone psychiatric units over GHPUs could regressively lead society back to a mental asylum era, ultimately undermining the progress made in mental healthcare (Duffy et al., 2019a).

## Ideological concerns

Another critique voiced by psychiatrists against IMHCA 2017 is of an ideological nature. Many in this field contend that the act heavily reflects Western principles of individual autonomy and human rights, which may not seamlessly align with the collective values of Indian society (Duffy and Kelly, 2019a; Math et al., 2019b). This divergence is particularly apparent in a collectivistic society like India. Some psychiatrists further argue that the Westernized legal approach endorsed by the act’s provisions for mental healthcare might not be an optimal fit for the Indian context (Duffy and Kelly, 2019a; Math et al., 2019b).

## Comparison of criticisms and concerns by psychiatrists in the light of CRPD guidelines

1. Psychiatrists have raised noteworthy criticisms regarding the IMHCA, particularly concerning provisions such as detailed documentation, stringent criteria for involuntary admission, the patient’s right to refuse treatment, advanced directives, regulation of electroconvulsive therapy (ECT) and the involvement of nominated representatives and mental health review boards. It is crucial to acknowledge that these criticized provisions align with the guidelines outlined in the CRPD. The CRPD preamble emphasizes the imperative to eliminate discrimination based on disability, highlighting the need to protect the human rights of all individuals with disabilities. Article 4 of the CRPD mandates that government bodies ensure and promote the complete realization of human rights for individuals with disabilities without any form of discrimination. Articles 5 and 12 underscore the principles of equality, nondiscrimination and equal recognition before the law. Considering these pivotal CRPD articles, the involuntary inpatient treatment of individuals with psychiatric conditions against their consent may be perceived as a violation of their fundamental rights. However, the IMHCA 2017 attempts to address these CRPD guidelines by maintaining stringent admission criteria, respecting the right to refuse treatment, and striking a balance between adhering to CRPD principles and practical psychiatric considerations. Additionally, the IMHCA 2017 includes provisions like advanced directives, nominated representatives and mental health review boards to safeguard the rights and dignity of individuals living with mental health challenges and uphold the tenets of Article 3 (respect for inherent dignity) and Article 12 (equal recognition before the law) of the CRPD.
2. Psychiatrists have raised concerns regarding the regulation of ECT under IMHCA 2017, which introduces significant restrictions on its practice. The act prohibits unmodified ECT, emergency ECT and ECT for minors. This prohibition aligns with the stance of the UN’s expert on torture, who considers unmodified ECT an unacceptable medical practice potentially amounting to torture. Such a position resonates with Article 15 of the CRPD, emphasizing the right to be free from torture or inhumane treatment. IMHCA 2017’s ban on emergency ECT aligns with the CRPD’s emphasis on informed consent for procedures with substantial long-term side effects. Articles 15 and 17 of the CRPD underscore the critical role of consent in medical decision-making and affirm the right to physical and mental integrity. Consequently, any medical intervention, including ECT administered without consent, may be perceived as a violation of the CRPD’s principles and guidelines.
3. Psychiatrists have raised a notable critique concerning the lack of clarity in certain definitions and terms within IMHCA 2017, with a specific focus on the assessment of capacity for individuals with mental health issues. This ambiguity, according to psychiatrists, poses a risk of generating uncertainty in clinical care and potentially fostering coercive treatment practices. The concern revolves around the possibility that both clinicians and nominated representatives could enforce involuntary treatments by demonstrating the absence of mental capacity based on clinical symptoms. Moreover, the act is criticized for inadequately addressing the concept of legal capacity in its provisions. In contrast, CRPD Article 12 explicitly asserts the right of individuals with disabilities to enjoy legal capacity on an equal footing with others, encompassing treatment decisions. A strict interpretation of CRPD Article 12 implies that any form of involuntary admission or treatment runs counter to its principles.
4. Psychiatrists have raised a significant critique of IMHCA 2017, pointing out the absence of provisions ensuring community-based mental healthcare, safeguarding social rights and eradicating stigma and discrimination against individuals with mental illness. In contrast, CRPD guidelines explicitly advocate for these rights, emphasizing community-based treatment and rehabilitative services as fundamental rights for individuals with disabilities in Article 19. Furthermore, Article 5 of the CRPD strictly prohibits discrimination on the grounds of disability, mandating states to ensure protection against discrimination across all spheres. Article 1 of the CRPD highlights the convention’s purpose in promoting, safeguarding and ensuring the full enjoyment of all human rights and fundamental freedoms for individuals with disabilities. Despite IMHCA 2017 acknowledging the significance of upholding the rights of individuals with mental illness in Section 18, the provided provisions are not as comprehensive as those outlined in the CRPD guidelines.
5. Psychiatrists express notable concerns regarding the feasibility challenges associated with the implementation of IMHCA 2017, emphasizing the prevailing human resource shortage, work culture, political will and infrastructure constraints within the Indian context. They argue that achieving the full implementation of the act, both in its intended letter and spirit, is contingent on substantial funding allocation and comprehensive manpower training. Importantly, these concerns align with the CRPD’s emphasis on the need for states to safeguard the rights of individuals with disabilities across all domains, including the fundamental right to health. In alignment with CRPD guidelines, Section 29 of IMHCA 2017 specifically assigns legal responsibility to the government for devising, formulating and executing initiatives to advance mental health promotion and prevent mental illness.
6. Psychiatrists have raised a notable critique against IMHCA 2017, specifically focusing on the perceived oversight of caregivers. This criticism revolves around provisions such as nominated representatives, advanced directives and certain limitations on family members’ rights, which are interpreted as lacking adequate consideration for caregivers. While acknowledging the pivotal role of family members in supporting individuals with mental illness, concerns are highlighted regarding instances of coercive behaviors exhibited by family members. The rights-based approach, guided by Article 12 of the CRPD, emphasizes the need to safeguard the autonomy and legal capacity of individuals with disabilities, extending this protection even against family members. The provision allowing the selection of representatives beyond the immediate family is seen as an attempt to uphold patient autonomy, especially in cases where mistrust exists. It is essential to note that the act does not mandate the selection of representatives from outside the family. Additionally, the inclusion of provisions for advanced directives is perceived as empowering patients, aligning with the principles of patient autonomy. While acknowledging potential financial burdens on family members, the state is urged to establish support systems. The new MHA underscores the importance of respecting the privacy of individuals with mental illness, in accordance with Article 22 of the CRPD. Thus, the exclusion of family members from accessing information without explicit patient consent is viewed not as a violation of rights but as a safeguarding measure guided by CRPD principles.
7. Psychiatrists express apprehensions regarding the perceived impact of IMHCA 2017 on the trust between doctors and patients, viewing provisions like informed consent, nominated representatives, advanced directives and mental health review boards as potential obstacles to fostering trusting relationships in mental healthcare. Nevertheless, the new MHA embraces a rights-based paradigm that prioritizes individual autonomy, aligning with the guidelines of the CRPD.
8. Psychiatrists express significant concerns regarding the potential repercussions of IMHCA 2017 on the existence of GHPUs. The apprehension stems from the act’s mandatory registration requirements, encompassing all voluntary admissions of adult individuals with mental health issues. This inclusivity may lead to the impracticality of accessing inpatient psychiatric care at unregistered GHPUs. The argument holds weight as the segregation of patients under this provision could intensify stigma and impinge upon the autonomy of individuals seeking mental health treatment, limiting their ability to choose inpatient care settings according to their preferences. Such constraints on patient autonomy may conflict with the principles advocated in the CRPD guidelines.

## Discussion

This scoping review thoroughly investigated psychiatrists’ viewpoints on the IMHCA 2017, revealing key themes. Limited global research exists on a comparable theme based on our current knowledge. A recent study from Saudi Arabia, which concentrated on mental health professionals’ perceptions of The Saudi Mental Health Care Law, included 46.06% psychiatrists among its 258 participants. The study reported a positive outlook, with 66.67% of participants agreeing that the legislation effectively ensures treatment for individuals requiring involuntary admission (Almadani et al., 2023). Our study yielded mixed results, as numerous psychiatrists opposed a shift toward a rights-based framework in the mental healthcare act, citing unique characteristics of the country. Some contended that in India, where doctor–patient relationships wield substantial influence in treatment decisions, systemic factors like illiteracy and cultural norms favoring paternalism make a stringent legal approach less practical. However, the new MHA embraces a rights-based paradigm, prioritizing individual autonomy, which aligns with the guidelines of the CRPD. Psychiatrists are called upon to be cognizant of the benefits and risks associated with the shift toward rights-based healthcare delivery. Embracing this new paradigm necessitates a substantial transformation in psychiatric practice, urging professionals to adapt to the evolving landscape and incorporate principles grounded in a rights-based approach. Moreover, to enhance the acceptability of the mental healthcare act among psychiatrists, addressing their concerns and providing education about the act’s philosophical and legal foundations is crucial. By analyzing criticisms through the lens of CRPD guidelines, targeted responses can be formulated. This not only helps in addressing specific concerns raised by psychiatrists but also contributes to the development of a legal framework that ensures alignment with international standards. Additionally, conducting educational programs can play a pivotal role in enlightening psychiatrists about the rationale behind the right-based shift, fostering a better understanding of the act’s objectives and encouraging its effective implementation.

Indian psychiatrists have articulated a spectrum of criticisms against the IMHCA 2017, reflecting a multifaceted landscape of concerns rooted in a paternalistic attitude, challenges to power dynamics and a perceived lack of understanding concerning the act’s right-based shift, particularly in the context of the CRPD guidelines.

One noteworthy critique focuses on the implications of specific provisions on psychiatric practice, including concerns about detailed documentation, stringent criteria for involuntary admission, the patient’s right to refuse treatment, advanced directives and the regulation of ECT. While these provisions align with CRPD guidelines, emphasizing nondiscrimination and the rights of individuals with disabilities, some psychiatrists, functioning within a paternalistic role in certain Indian contexts, raise questions about the practicality of a strict legal approach. They emphasize the importance of doctor–patient relationships, challenging the feasibility of implementing a stringent legal framework.

The regulation of ECT constitutes another layer of criticism, with concerns voiced about the prohibition of unmodified ECT and emergency ECT. While these measures align with CRPD principles, some psychiatrists argue against a complete ban, emphasizing ongoing debates among clinicians and the act’s right-based approach, particularly in banning emergency ECT. The tension between autonomy, informed consent and the potential for violations of CRPD principles becomes apparent in this critique.

Another significant critique revolves around the lack of clarity within the act, particularly concerning capacity assessments for mental health treatment decisions, aligning with CRPD guidelines. The identified gap in IMHCA 2017 regarding the definition and assessment of capacity requires rectification and there is a need to incorporate the CRPD’s concept of legal capacity. Psychiatrists express concerns about the potential for confusion in clinical care, the risk of coercive practices and the act’s failure to adequately address legal capacity issues. This critique underscores the imperative need for clarity in capacity assessments, aligning with Article 12 of the CRPD, especially during future amendments to the act.

The absence of provisions ensuring community-based mental healthcare, safeguarding social rights, and eradicating stigma and discrimination against individuals with mental illness represents yet another area of concern. While the act acknowledges the importance of upholding the rights of individuals with mental illness, it falls short of comprehensively addressing these issues outlined in the CRPD guidelines, leaving many critical rights concerns unaddressed. To align more closely with the CRPD guidelines, future amendments to the act must address these deficiencies and ensure a more robust framework for safeguarding the rights of individuals with mental health challenges.

Feasibility challenges tied to the implementation of the act constitute a substantial concern. Critics argue that achieving full implementation is contingent on substantial funding allocation and comprehensive manpower training, given prevailing challenges within the Indian context. However, the act, in alignment with CRPD guidelines, assigns legal responsibility for mental health promotion and prevention of mental illness to the government, emphasizing the need for increased government expenditure and resource allocation toward mental health. Given the legally binding nature of both the CRPD and IMHCA 2017, mental health professionals bear the mandate to advocate for increased government expenditure and resource allocation toward the treatment and rehabilitation of individuals living with mental illness. Leveraging the provisions of IMHCA 2017, this presents a pivotal opportunity to address the existing treatment gap in mental health and drive forward meaningful change.

Another substantial critique involves concerns about the perceived lack of consideration for caregivers. Provisions such as nominated representatives, advanced directives and certain restrictions on family members’ rights have been interpreted as indicative of insufficient regard for caregivers. However, it is crucial to recognize that these measures are aimed at empowering PwMI, and any exclusion of family members from accessing information without the explicit consent of the patient should be seen not as a violation of rights but as a safeguarding measure. This approach is intended to uphold the fundamental human rights of individuals with mental illness and is guided by the principles outlined in the CRPD.

A further critique revolves around the potential exacerbation of mistrust between patients and doctors. Provisions such as informed consent, nominated representatives, advanced directives and mental health review boards are viewed as potential impediments to fostering trusting doctor–patient relationships. However, the new MHA embraces a rights-based paradigm, prioritizing individual autonomy-based decision-making over paternalistic approaches, aligning with the principles outlined in the CRPD guidelines. In light of this shift, psychiatrists should be cognizant of both the benefits and potential risks associated with rights-based healthcare delivery. Embracing this paradigm necessitates a significant transformation in their practice, urging psychiatrists to adapt to the evolving landscape and incorporate the principles of the rights-based approach.

Finally, concerns have been raised about the potential consequence of GHPUs being forced to close due to mandatory registration requirements. The argument holds merit as such segregation of patients might exacerbate stigma and infringe upon the individual autonomy of patients with mental health conditions, restricting their ability to seek inpatient care at the setting of their preference. Such a constraint on a patient’s autonomy in selecting their treatment environment could potentially contravene the principles espoused in the CRPD guidelines. To address this issue, it is imperative that clear and well-defined exemptions be introduced in future amendments to the act, thereby fostering and preserving the viability of inpatient care within GHPUs for patients who willingly opt for such a care setting.

This results of this study holds far-reaching implications for mental health policy and practice. By aligning the IMHCA 2017 with international human rights standards, particularly the CRPD guidelines, this study contributes to a global discourse on refining legislation to protect the rights of individuals with mental health conditions. The critical appraisal offers valuable insights for policymakers, aiding in the identification of areas for improvement within the existing legal framework. Furthermore, the study facilitates global comparisons, highlighting regional variations in psychiatrists’ perspectives on mental health laws. It serves as a foundation for informed mental health practice, informing practitioners, educators and policymakers about the crucial role of mental health professionals in shaping legal and ethical considerations. The study’s implications extend to potential research avenues, emphasizing the need for comprehensive investigations into professionals’ viewpoints on legislation, ultimately advancing the broader human rights discourse in mental health.

## Strengths of the study

This study demonstrates strengths in conducting a comprehensive literature review across two major databases. It provides an in-depth exploration of psychiatrists’ perspectives on the IMHCA in light of CRPD guidelines, ensuring a nuanced understanding. The critical appraisal of the IMHCA in the context of CRPD guidelines contributes to the discourse on aligning mental health legislation with international human rights standards, offering valuable insights into potential improvements. Focusing on psychiatrists’ perspectives is crucial, as their insights play a pivotal role in shaping mental health practice and can inform policy discussions and implementation strategies.

## Limitations of the study

The study’s limitations include reliance on the existing published literature from limited research databases, potentially excluding firsthand perspectives or real-time data. This limitation may hinder the study’s ability to capture recent developments or evolving opinions. Findings are contingent on the availability and accessibility of published literature, introducing the possibility of publication bias, as unpublished or less accessible materials may not be adequately represented in the analysis.
