# Supplementary material for: Perceptions regarding the Indian Mental Healthcare Act 2017 among psychiatrists: Review and critical appraisal in the light of CRPD guidelines
Source: Glob Ment Health (Camb). 2024 Mar 19;11:e39. doi: 10.1017/gmh.2024.31 (PMC11016366; doi:10.1017/gmh.2024.31)
Supplement: Peer review history

# Editor report: Recommendation: Perceptions regarding the Indian Mental Healthcare Act 2017 among psychiatrists: Review and critical appraisal in the light of CRPD guidelines — R0/PR2

**DOI:** 10.1017/gmh.2024.31.pr2

**Affiliations:** Director, National Institute of Mental Health, Czech Republic

No accompanying comment.

# Editor report: Decision: Perceptions regarding the Indian Mental Healthcare Act 2017 among psychiatrists: Review and critical appraisal in the light of CRPD guidelines — R0/PR3

**DOI:** 10.1017/gmh.2024.31.pr3

**Affiliations:** Johns Hopkins University Bloomberg School of Public Health, United States

No accompanying comment.

# Editor report: Recommendation: Perceptions regarding the Indian Mental Healthcare Act 2017 among psychiatrists: Review and critical appraisal in the light of CRPD guidelines — R1/PR5

**DOI:** 10.1017/gmh.2024.31.pr5

**Affiliations:** Director, National Institute of Mental Health, Czech Republic

No accompanying comment.

# Editor report: Decision: Perceptions regarding the Indian Mental Healthcare Act 2017 among psychiatrists: Review and critical appraisal in the light of CRPD guidelines — R1/PR6

**DOI:** 10.1017/gmh.2024.31.pr6

**Affiliations:** Johns Hopkins University Bloomberg School of Public Health, United States

No accompanying comment.
